# Weaved into the cultural fabric*:* a qualitative exploration of alcohol consumption during pregnancy among tribal women in Odisha, India

**DOI:** 10.1186/s13011-018-0146-5

**Published:** 2018-02-20

**Authors:** Sanghamitra Pati, Abhimanyu Singh Chauhan, Pranab Mahapatra, Devraj Hansdah, Krushna Chandra Sahoo, Sandipana Pati

**Affiliations:** 1 Department of Health Research, Govt. of India, ICMR - Regional Medical Research Centre, Chandrasekharpur, Bhubaneswar, Odisha 751023 India; 2 Public Health Foundation of India, Plot 47, Sector 44, Gurugram, Haryana 122002 India; 3 Department of Psychiatry, Kalinga Institute of Medical Sciences, Campus 5, KIIT University, Bhubaneswar, Odisha 751024 India; 4 Department of Health and Family Welfare, Government of Odisha, Bhubaneswar, Odisha 751001 India; 5 Independent Research Consultant (Public Health), Bhubaneswar, Odisha 751001 India

**Keywords:** Alcohol misuse, Culture, Tradition, Tribal women, Pregnancy, Odisha, India

## Abstract

**Background:**

Evidence-based research has documented the association between alcohol intake during pregnancy and increased risk of miscarriage, stillbirth and congenital birth defects. Alcohol consumption is a complex behavior whose origins lay in cultural norms and the social structure. In tribal communities in India, alcohol misuse among women is a public health problem. This study is intended to explore perceptions and beliefs among tribal women and the community towards alcohol consumption during pregnancy.

**Methods:**

A qualitative study was conducted in a tribal-dominated district of Odisha, India. The WHO AUDIT tool was used to identify women who consumed alcohol during their pregnancies. In-depth interviews were conducted with 19 eligible women and 18 family members. Additionally, two focused group discussions were held with local community leaders and health workers. The data was transcribed, systematically coded and analyzed following the thematic framework approach.

**Results:**

The findings suggest that a complex interplay of drivers contributes to the unrestricted intake of alcohol by pregnant women. This could be attributed to: a lack of social monitoring, easy access to alcohol, low alcohol literacy and alcohol’s normative status in daily customs and traditions. Another contributing factor is a community-wide perception that home-made alcohol poses no ill effects.

**Conclusion:**

Alcohol consumption is deeply embedded in the daily rituals of indigenous tribal women. To address this issue, community counselling utilizing platforms of RMNCHA and VHND could be Ideal. A well-designed, culture-based intervention encompassing alcohol researchers, mental health specialists, public health workers and anthropologists is necessary.

## Background

The adverse effect of alcohol on a fetus or a child is well-acknowledged [1]. Alcohol consumption during pregnancy can cause miscarriage, stillbirth, and a range of lifelong physical, behavioral and intellectual disabilities known collectively as fetal alcohol spectrum disorders (FASDs) [2]. According to the Centers for Disease Control (CDC), no known amount of alcohol is safe to drink while one is pregnant. There is also no known safe time to consume alcohol during pregnancy, nor is there a safe type of alcohol to consume [3]. Evidence suggests that the consumption of even small amounts of alcohol during pregnancy can increase the risk of miscarriage, stillbirth, prematurity or sudden infant death syndrome [4].

Despite the existence of international guidelines advocating total abstinence from alcohol consumption during pregnancy, many developed countries have consistently reported a significant intake of alcohol by women during pregnancy [5]. This includes countries with intense awareness campaigns and high literacy rates. Most low- and middle-income countries report an increasing prevalence of alcohol misuse [6]. Some studies report that alcohol misuse is magnified in tribal communities [7]. Unlike in other communities, a substantial number of alcohol users in tribal communities are women [8]. If even a small proportion of these women continue to consume alcohol during pregnancy, the public health repercussions could be significant. This is especially significant given the poorer maternal and child health indicators prevailing in the tribal population [9].

A seemingly endless range of subgroups and individual variations among ethnic groups, as well as among related beliefs and practices, creates a new dimension with respect to the occurrence of phenomena or the transmission of disease in respective settings. These dynamics cannot be explained by biomedical pathways of diseases and associated factors. Tribal practices and their associated effect on health are well-documented [10, 11]. In a previous study, tribal people’s perceptions and practices affecting newborn health were reported in similar settings. The application of indigenously made substances to the baby’s umbilical stump and skin, the bathing of the baby immediately after birth, the late initiation of breast-feeding and ‘Budu practices’ were common. Newborns with small white or yellow cystic vesicles/spots in their palates were known as ‘Budu’ (wet) children and given frequent cold baths [12]. Unless the cultural beliefs underpinning traditional practices are known, any change in risky behavior contributing to ill health may be difficult to achieve. This is more applicable to a country like India, which has a diverse sociocultural structure, because each ethnic group maintains a system of practices related to its traditions and customs. This fact necessitates a community-specific study.

It is not yet known whether tribal women consume alcohol during pregnancy. Nor are their beliefs towards – and perceptions of – the potential effects known. The present study is the first of its kind aimed at exploring perceptions and practices related to the consumption of alcohol by pregnant tribal women in a tribal-dominated district of Odisha, India. The community reported use of *Handia/arrack* in their day to day customs and traditions. The drink has reported 20% to 40% of alcohol content [13].

## Methods

### Study setting and study design

**Fig. 1:**
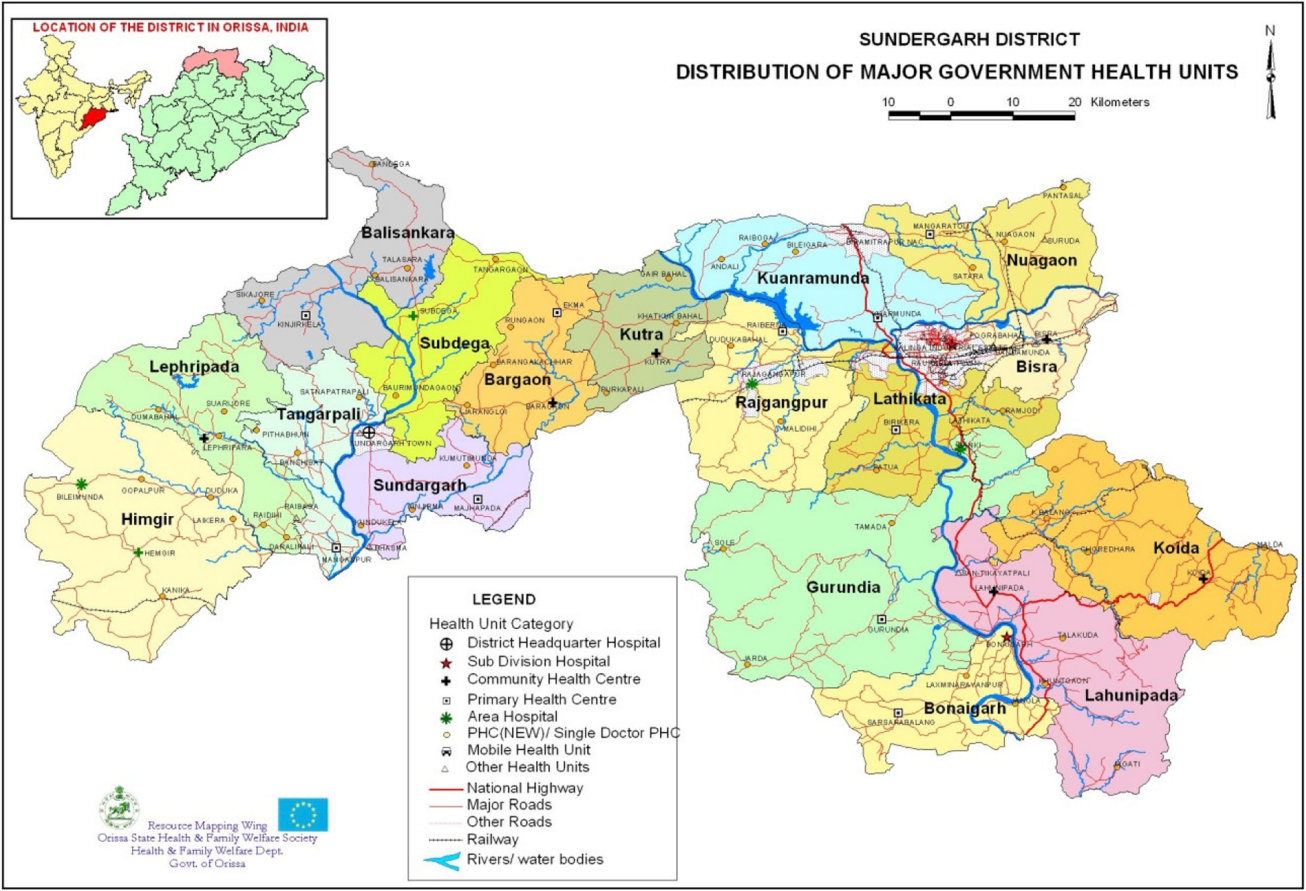
Map of Sundargarh district. The map shows the political boundaries of the various blocks under the Sundargarh block of the Sundargarh district of Odisha

The Sundargarh district is one of 30 districts within Odisha, and is located in the north. This district has a population of 2,080,664; tribal subgroups constitute around 51% of the total population (Fig. 1). A significant population of Santal and Munda tribes are found in the Sundargarh district [14, 15].

A qualitative study was carried out between June 2015 and July 2015 in the 29 villages of the Bisra community health centre (CHC) and the 12 villages of the Birkera CHC in the Sundargarh district. Because the study’s main objective was to understand the cultural aspect of alcohol use, participants were selected purposively and a qualitative inquiry was conducted.

### Study participants

The researchers employed the concept of cultural anthropology, involving emic and etic approaches towards understanding human behavior and related practices [16]. The study maintained an emic approach by evaluating the individual’s perspective and an etic approach by evaluating health system functionaries or a ‘culturally neutral’ perspective pertaining to the domain of inquiry. Records of women who had been lactating in the past three months were obtained from the two CHCs. The time frame was purposefully selected to create a recall period of 12 months. A short descriptive questionnaire was administered among these women primarily to learn about alcohol consumption practices during pregnancy, as well as to ascertain general demographic details related to age, education, ethnicity, family members who consumed alcohol, the child’s weight at birth, complications during birth, etc. The World Health Organization’s Alcohol Use Disorders Identification Test (AUDIT) was used to collect data on alcohol use. In a previous study, the tool had been validated in the local settings [17].

Women who reported having consumed alcohol during pregnancy were recruited as the key informants in this study. The family members of these women were also interviewed. To obtain the etic perspective, front-line workers of health systems (ASHA – Accredited social health activists, ANM – Auxiliary nurse midwives and AWW – Anganwadi workers) were also interviewed.

### Instrument and data collection

Structured thematic guides were developed for qualitative data collection using a combination of structured questions and pre-planned probes to create a detailed understanding of the issue. Guides were developed through an extensive review of previously published studies in a similar context across the globe and by consulting local experts in the domain to make the guides suitable for the regional context (Table 1). Face-to-face in-depth interviews (IDIs) were conducted in the local language by three authors of the study (DH, ASC and SP2) who were well-versed in qualitative data collection techniques and the local language. The research team comprised two female researchers (SP1 and SP2) and four male researchers (DH, ASC, PM and KCS). One of the authors (DH) belonged to the tribal community under study and was familiar with the local culture. DH visited the community prior to the start of the main data collection phase and established a rapport with the local community as well as with the local village leader. Interactions were scheduled at a time and place convenient for the participants. A trained facilitator moderated the focus group discussions with the health functionaries. Two other investigators helped conduct the focus group discussions (FGDs) with sociograms and the like. Both the FGDs were carried out at the government health facility. After five interviews, the interview guide was reviewed and revised to reflect emerging findings.

**Table 1:** Thematic guide

Stakeholders [Nos.]	Domains of Inquiry Sub-Themes
In-depth interviews with pregnant women [19]	1. Knowledge about alcohol • Composition of alcohol – How and when did you first start drinking alcohol? • Access – Where do you get alcohol? Who drinks in your family? • With whom do you drink alcohol?
	2. Practice and opinion about consumption • On what occasions or cultural activities do you consume alcohol? • What is your opinion about the consumption of alcohol (benefits and harms)?
	3. Alcohol and baby • What do you think the effect of alcohol would be on a baby (benefits and harms)? • On what occasions have you consumed alcohol since you conceived? • To your knowledge, why do women consume alcohol during pregnancy?
In-depth interviews with family members of pregnant women [18]	1. Practice and opinion about consumption • On what occasions or cultural activities do pregnant women consume alcohol? • What is your opinion about the consumption of alcohol (benefits and harms)?
	2. Alcohol and baby • What do you think the effect of alcohol would be on a pregnant woman and baby (both benefits and harms)? • On what occasions has the pregnant woman consumed alcohol since she conceived? • In your opinion, why do women consume alcohol during pregnancy?
Focus group discussion with front-line workers and community leaders. [two with 10 participants each]	1. Situation regarding alcohol consumption in the community • What do you think about alcohol consumption in this community? • Who consumes alcohol in the community? • Are there any special cultural gatherings and occasions on which alcohol consumption is promoted?
	2. Situation regarding the practice of alcohol consumption among pregnant women • Do you all know some women in your village who drink alcohol? Anyone who drinks during pregnancy? • Why do they drink? • Did any one of you ever counsel any pregnant women to not drink alcohol? (If yes, why and how? If no, why?)
	3. Knowledge and perception regarding the effect on alcohol on baby • Do you all think alcohol has an effect on an unborn baby? (If yes, what are they? If no, why not?) • Do you all think we should allow them to drink alcohol? [At this point, brief the participants about the ill effects of alcohol consumption on an unborn baby.] What is your suggestion in this situation? – how to combat the practice of alcohol consumption among pregnant women

An assistant recorded notes about the interviews and group interactions. The focus group discussions were carefully moderated to gather data regarding participants’ perceptions of alcohol use, the importance of alcohol in rituals, and alcohol use during pregnancy. Systematic procedures were employed to ensure reliability and validity in data collection. These procedures included verifying data with participants during and at the end of each interview and focus group, conducting a debriefing session between the moderator and assistant moderators immediately after each group, and using field notes and audio transcripts. All the interviews with the women and family members were conducted at their homes, while the FGDs were conducted at a nearby government facility. Due to limited resources and time, repeat interviews were not conducted, nor was feedback sought on findings. In-depth interviews lasted between 30 and 40 min. Most – but not all – the interviews were conducted in private. Some participants requested the presence of family members, and the researchers agreed to this request. IDIs and FGDs were digitally recorded and transcribed to maximize data capture and facilitate analyses. All the verbatim transcriptions were translated to English by a professional agency. The investigator randomly checked 10% of the recordings to ensure the quality of the transcription.

### Data analysis

To identify themes and subthemes, the qualitative interviews were analyzed using an inductive approach that included familiarization with the data, the identification of a thematic framework, and the charting, indexing, mapping and interpretation of the data. Two investigators (SP1, ASC) conducted initial coding independently. Once the transcripts were read and coded, they met to discuss the open coding. Through extensive discussion, they determined a series of thematic codes that, through axial coding, described agreed-upon categories and subcategories. Axial coding was further narrowed down to selective coding. ATLAS.ti version 7 was used for data management.

## Results

Two CHCs reported a total of 132 women lactating in the past three months in the 41 villages under their jurisdiction. Out of these 132 women, 119 agreed to participate in the first round of the survey. Twenty women reported consumption of alcohol during pregnancy. Sixteen (80%) out of these 20 women scored 3 on AUDIT screening questionnaire (positive or optimal for identifying hazardous drinking or active alcohol use disorder). Four women scored 4 on the scale [17]. All reportedly belonged to tribal communities (Santal and Munda) in which *handia* consumption was reported. Five (20%) women interviewed were illiterate. Seven (35%) reportedly belonged to the Below Poverty Line (BPL) category. Low-birth-weight babies were reported by 13 (65%) women.

Out of these 20 women, 19 agreed to participate in the qualitative inquiry. The data saturation was reached by the 19th interview. Similarly, 18 family members were interviewed. Two focus group discussions with 10 participants each were also conducted with relevant front-line workers and community leaders.

Through the thematic framework analysis, four categories of drivers affecting the consumption of alcohol during pregnancy emerged: A. Customs, tradition and rituals; B. Indigenous, non-injurious and relaxant; C. Family and peer support and easy availability; and D. Curiosity, addiction and lack of knowledge.

A. *Custom, tradition and rituals*

The Odisha tribe observes a string of festivals. Some are closed affairs, relating to a birth or death within the family. Others relate to sowing or harvest time and involve the entire community. Whether the occasion is a birth or a death, in the Santal and Munda communities, “*handia”* is offered as a means of worshipping the god and goddess *and* is consumed at the end of worship as *prasad (*part of the edible offered to the god). For all stakeholders, including women, family members, frontline workers and community leaders, *handia* occupies the culture and tradition. They believe that gods and spirits, particularly the malevolent ones, must be appeased first so that they do not unleash drought or sickness on the land. Most fathers-in-law, mothers-in-law and community leaders stated that *‘handia’* is an essential component for performing rituals at births, during festivals, during marriages, etc. Also, when one goes to a friend’s or relative’s house, *handia* is offered as a gift. This act shows the visitor’s status, love and affection. Similarly, the host greets visitors with *handia*.

*“It’s our tradition. We drink alcohol because it is an essential item in all rituals, may it be birth celebration, death mourning or festival.”* (IDI 04, Mother-in-law, 61)

*“My husband and all family also drinks alcohol. So I also drink alcohol. We drink alcohol as part of our life style, eating and drinking during bonga (worship)*. *I am drinking handi since my childhood as prasad and I like it too much.”* (IDI 01, Woman, 26)

B. *Indigenous, non-injurious and relaxant*

Most of the women and mothers-in-law considered *handia* a safe drink, as it is made only of rice. They consider commercial alcohol to be intoxicating and injurious to health, but not the homemade *handia.* Most of them said that *handia* acts as a relaxant, giving pleasure and enabling one to forget all the sorrows and anxieties of life. A few women said that family members, including their mothers-in-law and husbands, advised them to drink *handia* during pregnancy because it could possibly alleviate abdominal pain and help with cramping.

*“Do you know the ingredient of the handia? It’s not the same which is being sold at the beer shop. We make it in our house using only rice. A good quality rice. Why would it be harmful? It’s like food.”* (IDI 01, Mother-in-law, 59)

*“They (husband and mother-in-law) asked me to drink handia when I was in first trimester and suffering from pain in stomach.”* (IDI 07, Woman, 32)

C. *Family and peer support and easy availability*

Nearly all women reported that most of the time family members, including husbands, mothers-in-law, fathers-in-law, etc., supported the intake of alcohol. A few women reported that they received encouragement from seeing all family members consume *handia*. Additionally, due to the daily use of *handia* in most household activities, non-availability was never an issue. At any time, plenty of *handia* was available in the household for consumption. A woman who was addicted did not have to purchase it from the market.

*“It’s not considered as taboo, we all drink together whether it’s with husband or parents-in-law.”* (IDI 02, Mother-in-law, 62)

“*See, it is being used whole day, during daily worship, greeting the guests coming in your house etc. We keep making it on continuous basis, like we cook food. So we have enough whenever we want to have.”* (IDI 15, Woman, 21)

D. *Curiosity, addiction and lack of knowledge*

Many women reported that curiosity was the reason behind their first experience with alcohol. Nearly all of them grew up seeing their family members habituated to *handia.* With no social control over intake, as reported by them, they slowly became habituated themselves*.* Women, family members and community leaders reported that most families did not consider alcohol to be injurious to health, and that some considered it to be good for one’s health. This information was transferred from generation to generation and resulted in *handia*’s traditional reputation as a healthy drink.

“*People here in this community is not aware about the bad effects of the alcohol to the body. Even if people are aware, they do not link with the handia as its part of their culture and tradition. Very few have knowledge related to ill effects of handia to fetus and they often try to make this understand to women and community.”* (FGD, Community leader, 58)

*“It’s a mix of two things, children do see their family members drink handia since beginning. They are curious about it and since there is no social boundation, most of them start having it from young age. Also people are not aware about its bad effects on different organs and to fetus if a pregnant lady consumes it. Situation is difficult to handle.”* (FGD, ANM, 45)

**Fig. 2:**
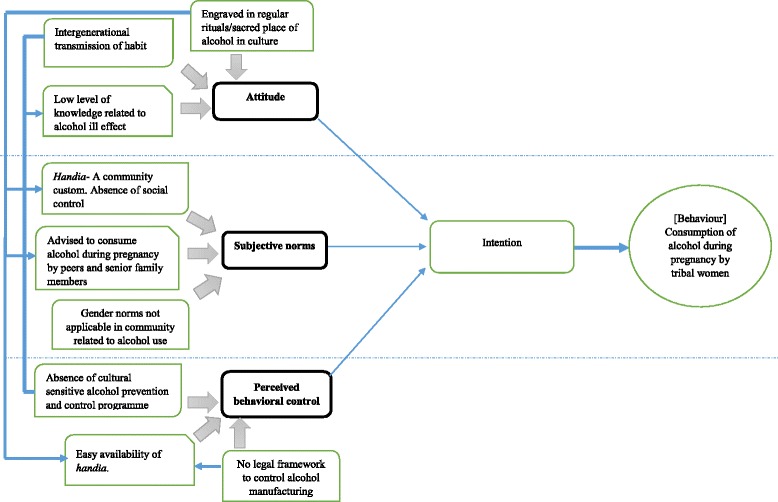
Conceptual framework explaining the interplay of factors contributing to alcohol intake during pregnancy. Using the theory of planned behaviour, the diagrammatic representations explain the various drivers of alcohol consumption among tribal women. This includes attitudes, subjective norms and behavioural control

The researchers explained the practice of alcohol consumption during pregnancy using the theory of planned behaviour [18]. According to this theory, explained in the context of the current issue under discussion, three factors affected the intention to consume alcohol: women’s attitudes towards the specific behavior, their subjective norms and their perceived behavioral control (Fig. 2).

E. *Challenges for the frontline workers*

Frontline workers shared their experiences related to counselling the pregnant women and their family members regarding no use of handia during pregnancy. Among these women, none of the frontline worker observed noticeable change realted to alcohol consumption behaviour. Most of thefrontline workers believed that handia strongly holds a sacred stature in the society since ages. Therefore its difficult to make women and family members understand the potential ill effects on the baby. Few frontline workers also reported that low education level in this particular tribal community also contributes to failure of any counselling and intervention.

“*See, these people are very spiritual, for them god is everything and handia is a spiritual drink. They won’t give away the drink for complete 9 months when they are in practice of consuming it on daily basis. Also, its not easy to make them understand biomedically that it can affect the child. Most of them didn’t attended school even once.We tried at our level in past but nothing has changed”* (FGD, ANM, 37)

*“No major community awareness programme have ever been implemented in this community related to prevention of alcohol abuse. Not by non-government organization”* (FGD, AWW, 42)

## Discussion

When investigating alcohol-related behavior and promoting abstinence from alcohol, one must remain attentive to the fact that processes that benefit the ethnic population in one context may be neutral, or even deleterious, in another. Recognizing a sensitivity to the need for heterogeneity, a number of published qualitative studies have advocated a culturally embedded understanding of health behavior. In this study, culture is understood as the customs, traditions, languages and social interactions that provide identity conclusions for individuals and groups. Context, as distinct from culture, is the social, temporal and geographic location in which culture is manifested. Few, but diverse, opinions about the impact of alcohol on an unborn child were received. Most of these opinions reflected perceived benefits. This indicates the existence of a space, albeit small, that could be further explored for sensitization and behavioral intervention.

### Intergeneration transmission of behaviours

Althoug the study is based on qualitative methods and a causal relationship cannot be drawn but the findings support use of handia in daily customs and traditions as value socialization, offering handia to guest as status inheritance and learning from family members as role modeling all are important mechanisms in understanding intergenerational transmission of similarities in family-behavioural patterns. Role modeling has been reported in past as an important mechanism underlying the intergenerational transmission of family-life behavioural patterns. In particular, if a parent and a child have lived together from each other during part of childhood, family behavioural patterns are more likely to be similar. The intergenerational transfer of is most likely be the case in the current study as the tribal communities lives as a closet society with least interaction outside and children mostly lived with their parents throught. Similarly, status and *Handia* is also linked to each other in the current context. The guest are offered with *Handia* during social gathering etc. Its linked to the status of the household. The generations most likely carries this status inheritance as intergeneration transmission. Simmilarly, each generation living with a close nit with the parents undergoes value socialization. In this context, we undertood that a person, from birth through death, is taught the place of *handia* in their norms, society and customs.

### Interplay of limited information and family/peer support

Despite increasing public awareness of antenatal care, many pregnant women in this population might consume alcohol to varying degrees. This is worrisome because these women prepare alcohol at home without adequate information. Thus, the concentration of this un-distilled alcohol is not monitored. As reported in this study by government functionaries, the situation becomes more complex when the population has a low literacy rate and when alcohol is part of the population’s religion and rituals because of customs and traditions. The usual notion of “home food” as synonymous with “benign and beneficial” labels alcohol as body-friendly. The fact that home foods, being indigenous, are not perceived as harmful could be one of the reasons why alcohol is regarded as benign and maintaining benefits that outweigh perceived harm among pregnant women.

Most people form their opinions based on upbringing, peer pressure, and personal, cultural and societal bias. Informal social control was reported as being an effective control measure engaging parents and relatives to be more active in monitoring activities. This may be related to lower levels of drinking [19]. However, women in this study reported that family members suggested they drink alcohol as a means of relieving pain during pregnancy. In this context, the opportunity for informal social control or norms is non-existent. This has resulted in the lack of an intervention point to prevent and control the use of alcohol during pregnancy.

A nearly similar finding was reported in previous studies, which found that alcohol consumption was considered a strategy for coping with stressors and negative emotions, including those associated with pregnancy [20]. Earlier studies found that physicians’ counselling resulted in a reduced intake of alcohol during pregnancy [19]. Similarly, in other parts of the world, ongoing programs exist for the prevention of fetal alcohol syndrome (FAS) and other alcohol-related birth defects (ARBDs); these programs can be modified and adapted to the local cultural context [21, 22]. Maternal health programs (ANC) in tribal populations should be cognizant of this hidden yet prevalent phenomenon that might have repercussions in terms of adverse fetal outcomes.

### Lack of policy and programmes

Several researchers have proposed that help-seeking is a process embedded in an individual’s social network and includes a series of choices. The three primary stages of help-seeking are: (a) problem recognition, (b) the decision to seek help and (c) service selection and utilization [23]. Movement through this help-seeking process is often complicated by multiple factors that may include early pregnancy, low education, migration to new places and a strange environment. When applied to our study population, the intersection of these obstacles, coupled with the status – and lack – of a health services policy for alcohol use among pregnant women compounds this social problem. While the West advocates complete abstinence from alcohol during pregnancy, no such policy exists for Indian women...

South Asian culture views alcohol consumption as a masculine attribute, and disapproves of women’s consumption [24]. This could be a marker of the subjective norms and gender hierarchy the supremacy being reflected though alcohol use. The gender norms posit that males have a natural tendency to drink, while for women, alcohol consumption is considered rebellious or is stigmatized. Because prevailing cultural norms and beliefs “in general” posit that women in South Asia do not consume alcohol, policy makers have viewed this act as a relatively unimportant public health issue for the female population as compared to other risky behaviors, like the use of smokeless tobacco. This gender bias has not infiltrated tribal communities under study, which are female-dominated societies. Unlike mainstream society, the gender hierarchy in tribal cultures is revered, and women maintain superior positions with respect to decision making and family matters. This could be another factor contributing to the perception of alcohol use as natural or normal among tribal women.

“Ideal” cross-cultural studies start with “local phenomenological descriptions.” Instead of assuming the nature of psychopathology from a culturally distant perspective, native accounts are essential to providing an understanding of it in local cultural contexts [25]. Knowledge of local perspectives has strengthened community-based interventions and improved and fostered health-seeking/healthy behavior. In the case of alcohol use by tribal women in our study, an intergenerational transmission exists of cultural beliefs and attitudes. Borrowing from social learning theory, norms, attitudes, expectations and beliefs arise from interactions with one’s cultural environment [26]. In tribal culture, women’s alcohol consumption is a socially embedded process, in which affiliation matters. The overall calculus of social exchange can configure the pattern of drinking behavior. Predominantly, this is irrespective of whether or not a woman is pregnant.

### Limitations

The study makes a unique, qualitative contribution to evidence-related alcohol use among tribal communities in India. However, it has some limitations. First, a small number of participants was interviewed in this study – a group that does not represent the population of the country’s larger tribal community. Second, all FGD and IDI with patients were performed in the local language, specific to the tribal communities under study, and then translated into English. Despite the rigorous verification process, some subtle nuances might have been missed during the verbatim transcribing.

## Conclusion

Tribal women and family members perceive alcohol as a part of their routine lives, and therefore continue consuming alcohol during pregnancy. The practice can be attributed to community’s limited knowledge about ill effect of alcohol on new born. The alcohol risk communication should be included in the current reproductive, maternal, newborn and child health, adolescent program (RMNCHA) [27]. Considering the complex dynamics of alcohol engraved in a community’s customs and traditions, wide community acceptance and absence of local context specific programme, it is suggested that Village Health and Nutrition Day (VHND) could be harnessed for community counselling [28].
